# Bioactive Potential of Olive Leaf By-Product Throughout *In Vitro* Gastrointestinal Digestion

**DOI:** 10.3390/foods14040563

**Published:** 2025-02-08

**Authors:** Mónica Sánchez-Gutiérrez, Ricardo Gómez-García, Elena Carrasco, Alejandro Rodríguez, Manuela Pintado

**Affiliations:** 1 Departamento de Ciencia y Tecnología de los Alimentos, UIC Zoonosis y Enfermedades Emergentes (ENZOEM), Campus de Excelencia Internacional en Agroalimentación (CeiA3), Universidad de Córdoba Rabanales, Edificio Darwin-Anexo, 14071 Cordoba, Spain; bt2cajie@uco.es; 2 Laboratório Associado, Escola Superior de Biotecnologia CBQF—Centro de Biotecnologia e Química Fina, Universidade Católica Portuguesa, Rua Diogo Botelho 1327, 4169-005 Porto, Portugal; rgarcia@ucp.pt (R.G.-G.); mpintado@ucp.pt (M.P.); 3 Grupo Biopren (RNM940), Departamento de Ingeniería Química, Instituto Químico para la Energía y el Medioambiente (IQUEMA), Facultad de Ciencias, Campus de Excelencia Internacional en Agroalimentación (CeiA3), Universidad de Córdoba, 14071 Cordoba, Spain; q42ropaa@uco.es

**Keywords:** functional food, phenolic compounds, antioxidant activity, *in vitro* digestion, agri-food wastes

## Abstract

Olive leaf, an abundant and underutilized byproduct of the olive industry, has gained attention as a potential functional ingredient due to its high content of dietary fiber and phenolic compounds. However, little is known about its bioaccessibility and transformation throughout the digestive process, limiting its application in food formulations. This study provides a comprehensive and quantitative assessment of how ground olive leaf bioactive compounds behave during gastrointestinal digestion, offering new insights into their stability and potential health benefits. The total phenolics content and antioxidant activity of ground olive leaf increased in the oral and gastric phases, decreasing slightly in the intestinal phase, with a bioaccessibility of 46% and up to 70% for the total phenolic content and antioxidant activity, respectively. The principal individual phenolic compounds identified in the intestinal phase were oleuropein, luteolin-7-glycoside, luteolin-6-glycoside and ferulic acid, with bioaccessibilities of up to 97%. The main soluble sugars (fructose, glucose, and sucrose) and organic acids (succinic, citric, and acetic acids) detected in the olive leaf samples showed different behaviors during gastrointestinal digestion: sugars increased in the oral and gastric phases but decreased in the intestinal phase, with high bioaccessibility despite reduced recovery, while organic acids remained mostly stable, except for citric acid, which decreased significantly in the intestinal phase, all showing close to 100% bioaccessibility. These results provide the first detailed evidence of the digestive fate of ground olive leaf bioactive compounds, reinforcing its potential as a functional ingredient. Its natural availability, without requiring pre-treatment, combined with its high antioxidant potential and bioaccessibility, highlights its relevance for the development of innovative food ingredients, aligning with circular economy principles and sustainable food strategies.

## 1. Introduction

Agriculture and the agri-food industry generate a large volume of non-edible parts resulting from agricultural production and processing [1,2]. Annually, up to 1.3 billion tons of biomass are generated from agricultural by-products, causing serious environmental pollution, as well as economic costs for the industries involved [3,4]. Spain is the world’s largest producer of olive oil. In the Andalusian region alone, olive harvests generate more than 4.5 million tons of by-products per year, of which around half a million comprises olive leaves, which have no further use [5]. The valorization of olive residues as natural materials rich in bioactive compounds could be a solution or alternative to the loss of natural resources. However, their current limited use contrasts with their promising applications in the food, pharmaceutical and nutraceutical industries [6]. The valorization of these olive residues, which are rich in bioactive compounds, presents a promising solution to the loss of valuable natural resources and aligns with the principles of the circular economy [7,8].

In recent decades, there has been a growing interest in moving towards more sustainable and environmentally friendly food production. This approach is an alternative to the current ‘take–make–dispose’ linear production model and is based on the need to find a sustainable use for all components of renewable resources with the aim of “zero” waste production [9,10]. In this sense, the valorization of olive residues, such as olive leaves, which are rich in polyphenols and dietary fiber, becomes essential to promote a green solution for the olive industry; the development of new ingredients from these residues is a key challenge for modern society [11,12]. In this scenario, together with the market trend for more sustainable food products and due to social awareness about the close relationship between diet and health, there is a greater demand by consumers for natural and functional food products with health benefits such as disease prevention [8,13,14,15]. In general terms, “a functional food is considered to be a food or beverage that can be consumed as part of the daily diet providing benefits beyond the nutritional function, i.e., enhancing a biological property or aiding in the prevention of disease” [16,17].

Olive leaf, one of the main by-products of olive and olive oil production, is a source of bioactive substances containing a significant amount of dietary fiber, mainly composed of cellulose and lignin (insoluble dietary fiber), and hemicellulose (soluble dietary fiber). In addition, olive leaf contains phenolic compounds that are bound to dietary fiber [18,19,20], which have been reported to exert antioxidant, antimicrobial and prebiotic activities [11,21,22,23]. The main phenolic compound present in olive leaf is oleuropein, an ester of hydroxytyrosol with elenolic acid, which is additionally β-glycosylated. A wide range of beneficial health effects have been previously attributed to olive leaf polyphenols, including antihypertensive, hypocholesterolemic, hypoglycemic, cardioprotective and anti-inflammatory effects [24].

While the composition and bioactive potential of olive leaf extract have been widely reported [25,26,27], limited research has focused on the bioaccessibility and transformation of these compounds throughout the gastrointestinal tract (GIT) [28,29,30,31], especially for ground olive leaves as a functional food ingredient.

Bioaccessibility can be defined as the proportion of a food component that is released from the food matrix during digestion and is available for absorption in the intestine and transferred into the bloodstream [32]. Thus, the positive health effects of consuming food ingredients of plant origin depend on the bioaccessibility of their bioactive components in the GIT [33]. Although such benefits have been widely reported, many phenolic compounds do not reach the gut as they are degraded along the GIT. In this regard, dietary fiber can protect phenolic compounds from oxidative degradation but also reduces their bioaccessibility by restricting the action of enzymes in the GIT [18]. Owing to the importance of predicting the bioaccessibility of bioactive molecules, the *in vitro* digestion method has emerged as the best alternative to *in vivo* studies as it is a simple, cost-effective and useful tool that simulates the passage of food components through the GIT by mimicking oral, gastric, and intestinal conditions [34].

This study enhances our understanding of the bioactive compounds in ground olive leaf and explores its potential as a functional food ingredient. In this context, the aim of this research was to evaluate the impact of *in vitro* gastrointestinal digestion on the bioaccessibility of ground olive leaf compounds by measuring changes in the composition of bioactive compounds and antioxidant activity of the soluble digested fraction.

## 2. Materials and Methods

### 2.1. Chemicals and Reagents

AAPH (2,2-azo-bis-(2-methylpropionamidine) dihydrochloride), ABTS diammonium salt (2,2-azino-bis (3-ethylbenzothiazoline-6-sulphonic acid)), anhydrous sodium carbonate (Na_2_CO_3_), fluorescein (F-6377), Folin–Ciocalteu’s reagent, hydrochloric acid (HCl), and sodium hydroxide (NaOH) were all acquired from Merck (Algés, Portugal). α-amylase, bile salts pancreatin, pepsin, sodium hydrogen carbonate (NaHCO_3_), calcium chloride dihydrate (CaCl_2_•2H_2_O), magnesium chloride hexahydrate (MgCl_2_•6H_2_O), ammonium acetate, *D*-(+)-glucose, potassium dihydrogen phosphate (KH_2_PO_4_), sodium hydroxide (NaOH) and sulphuric acid (H_2_SO_4_), as well as standards of Trolox (6-hydroxy-2,5,7,8-tetramethylchroman-2-carboxylic acid), gallic acid, caffeic acid, isoferulic acid, ferulic acid, 4-hydroxybenzoic, protocatechuic acid and vanillic acid were acquired from Sigma-Aldrich (Sintra, Portugal). Hydroxytyrosol, tyrosol, vanillin, myricetin, apigenin-7-glycoside, apigenin, oleuropein, luteolin, luteolin-7-glycoside, luteolin-6-glycoside and tyrosol were purchased from Extrasynthese (Lyon, France). Fischer Scientific (Oeiras, Portugal) provided the methanol and formic acid (Oeiras, Portugal).

### 2.2. Olive Leaf Material

Olive leaves of the “Hojiblanca” variety were collected from an olive grove in the province of Cordoba (Spain) just after the end of the olive harvesting season, in mid-March 2023. The prunings were immediately taken to the laboratory, where leaves were removed from the branches, manually washed and dried in the open air in the absence of light for 5 ± 2 days, until the leaf moisture was below 8%. They were then ground into powder in a Retsch SM2000 automatic grinder (Restsh GmbH, Haan, Germany) and sieved manually with an ASTM No. 10 sieve to obtain particles with a diameter of <2 mm. The ground olive leaves, with a moisture content of 5.17 ± 0.02%, were kept at room temperature in a dry and dark room before use.

### 2.3. In Vitro Simulated Gastrointestinal Digestion and Experimental Design

With some modifications, the approach previously reported by Madureira et al. [35] was used to investigate the impact of the *in vitro* simulated GIT on the stability of ground olive leaves (OL). Two grams of OL were dissolved in twenty milliliters of ultra-pure water to prepare the initial (undigested) samples. A dialysis procedure was used to replicate intestinal absorption, while several enzymes were used to mimic digestion. Prior to use, all enzyme solutions were prepared immediately and stored in ice until they were added. The temperature of the human body was replicated using a water bath set at 37 °C, while mechanical agitation at intensities comparable to those seen *in vivo* in each digestive compartment was utilized to mimic peristaltic motions. Aliquots of the digestion mixes were taken at the conclusion of each GIT phase (oral, stomach, and small intestine), frozen, and kept until their bioactive components and antioxidant activity were analyzed. Antioxidant activity (as determined by the ABTS and ORAC tests) and total phenolic content (TPC) were assessed both prior to, and during, exposure to the simulated digestion conditions.

First, the initial pH of the samples was adjusted to between 5.6 and 6.9 using 0.1 M of NaOH. Oral digestion was carried out by incubating the samples at 37 °C and 180 rpm for one minute after adding 0.6 mL of α-amylase solution (100 U/mL). Then, 1 M HCl was used to lower the pH of the samples to 2.0 for gastric digestion. After adding 25 mg/mL of pepsin at a rate of 0.05 mL/mL per sample, the mixture was incubated for one hour at 37 °C and 130 rpm in a shaking water bath. Then, 1 M NaHCO3 was used to lower the pH to 6.0 in order to carry out small intestine digestion. By adding a solution of pancreatin (2 g/L) and bile salts (12 g/L) at a concentration of 0.25 mL/mL per sample, the intestinal juice was mimicked. A long intestinal digestion was simulated by incubating the solution mixture for two hours at 37 °C and 45 rpm. Samples were moved into a cellulose acetate dialysis tube with a molecular weight cut-off of 3 kDa (Spectra/Pro, Spectrum Lab, Breda, the Netherlands) during the final stage of intestinal digestion in order to replicate the small intestine’s natural absorption step. The membranes were then submerged for 24 h at room temperature in a water bath that was periodically replaced and agitated at 1000 rpm. The amount of the sample that could be absorbed and enter the bloodstream was represented by the solution that was able to diffuse the dialysis tubing at the end of the procedure, whereas the non-absorbable sample (colon-available) was represented by the solution that remained inside the dialysis tubing. The experiments were conducted independently in three biological trials. During each digestion phase, the biological replicate was sampled in duplicate or triplicate, depending on the assay.

### 2.4. Bioaccessibility and Stability of Bioactive Compounds from OL Through In Vitro Gastrointestinal Digestion

#### 2.4.1. Recovery and Bioaccessibility Index

Two distinct percentage indices, the recovery index (RI) and the bioaccessibility index (BI), were examined in order to assess the impact of *in vitro* digestion on the phenolic compounds, organic acids, and sugars of OL [36,37]. The RI measures the percentage of bioactive compounds present in the digested food material after mouth, gastric, or intestinal digestion, according to the following equation:

(1) $$\;Recovery\;Index\left(RI,\%\right)=\frac{BC_{DS}}{{BC}_{TS}}\times 100$$

where BC_DS_ is the bioactive content (mg/100 g DM) in the digested sample at a specific gastrointestinal phase and BC_TS_ is the bioactive content (mg/100 g DM) quantified in the test sample (undigested). Despite potential GIT responses, bioactive chemicals must be liberated from the food matrix and retain their bioactive form in order to have an effect, or to be bioavailable. Bioavailability includes the bioaccessibility concept. The bioaccessibility index (BI) measures the proportion of solubilized bioactive substances recovered after the intestinal dialysis phase that can be available for absorption into the bloodstream (Equation (2)), as follows:

(2) $$Bioaccessibility\;Index\left(BI,\%\right)=\frac{BC_{Dy}}{{BC}_{IDS}}\times 100$$

where BC_Dy_ is the bioactive content (mg/100 g DM) absorbed after dialysis and BC_IDS_ is the bioactive content (mg/100 g DM) in the intestinal digested sample.

#### 2.4.2. Total Phenolic Content (TPC)

The Folin–Ciocalteu method, with minor modifications, was used to determine the TPC of OL in the various stages of *in vitro* gastrointestinal digestion [38]. In a 96-well plate, 20 μL aliquots of the samples were combined with 100 μL of 7.5% *w*/*v* sodium carbonate and 80 μL of the Folin–Ciocalteu reagent that had been previously diluted (1:10 *v*/*v*) with water. A multi-detection plate reader (Synergy H1, Santa Clara, CA, USA) was used to measure the absorbance at 750 nm following a one-hour incubation period at room temperature in the dark. The reference standard used was gallic acid. Results were expressed as mg gallic acid equivalents (GAE)/100 g DM. For every experiment, each measurement was made in triplicate.

### 2.5. Antioxidant Activity Determination

#### 2.5.1. ABTS Assay

The ABTS scavenging activity assay of OL was determined using a method previously described by Ribeiro et al. [39] for the different phases of *in vitro* gastrointestinal digestion. In brief, aliquots of samples were placed on microplates and a solution of ABTS and potassium persulfate reagents was added. The samples were then incubated for five minutes at 30 °C. An absorbance reader (Synergy H1, Santa Clara, CA, USA) with several detections was used to measure the values at 734 nm. Trolox standards were used to construct a calibration curve; the results were reported as mmol of Trolox equivalents (TE) per 100 g DM. For every experiment, each assay was run in triplicate.

#### 2.5.2. Oxygen Radical Absorbance Capacity (ORAC)

The ORAC assay was carried out using the procedure previously described by Ribeiro et al. [39]. The oxidation of fluorescein by peroxide radicals generated in situ by the thermal breakdown of AAPH is the basis of this technique. To create a calibration curve, serial dilutions of the antioxidant acid Trolox were prepared as a positive control. First, 120 µL of fluorescein (70 nM) was preincubated with 20 μL of phosphate-buffered saline solution (PBS, 75 mM, pH 7.4) containing Trolox or samples for 10 min at 37 °C in a black polystyrene 96-well microplate (Nunc, Denmark). Using a multichannel pipette, 60 µL of the AAPH solution (12 mM, final concentration) was added promptly. Fluorescence measurements were conducted using a multi-detector plate reader (Synergy H1, Santa Clara, CA, USA) for a period of 140 min at an excitation wavelength of 485 nm and an emission wavelength of 520 nm. Every day, solutions of AAPH and Trolox were prepared, and fluorescein was diluted in PBS from a stock solution (1.17 mM). All samples were prepared in duplicate and three independent trials were performed for each sample. The final ORAC values were expressed as mmol of Trolox equivalent (TE) per 100 g DM.

### 2.6. Identification and Quantification of Phenolics by HPLC

With minor modifications, the method described by Campos et al. [40] was used to determine the polyphenolic profile of OL acquired in each phase of the GIT using high-performance liquid chromatography with a diode-array detector (HPLC-DAD). Samples were introduced into an HPLC-DAD-interfaced Waters Series e2695 Separation Module System (Mildford, MA, USA). Two mobile phases, consisting of mobile phase A (water:methanol:formic acid, 92.5:5:2.5, %*v*/*v*/*v*) and mobile phase B (methanol:water:formic acid, 92.5:5:2.5, %*v*/*v*/*v*), were used to perform separation in a reverse-phase column (COSMOSIL 5C1 8-AR-II Packed Column—4.6 mm I.D. × 250 mm, Dartford, UK). The gradient and conditions were as follows: 50 μL of the sample was injected; the flow was continuous at 0.5 mL/min; the gradient elution began at 100% mobile phase A for 50 min and was then reset at 45% A and 55% B between 50 and 55 min; it then returned to 100% mobile phase A and remained there for 4 min (until 59 min). Empower 3 software was used to collect and analyze the data. Several compounds, such as catechins or procyanidins (280 nm), phenolic acids (320 nm), and flavanols (330 nm), were detected at wavelengths between 200 and 600 nm. These compounds were identified and measured using a calibration curve with pure standards in terms of retention times, UV absorption spectra, and peak areas at the maximum absorption wavelength. Every decision was made three times. The findings were reported as milligrams of phenolic compounds per 100 g of DM.

### 2.7. Identification and Quantification of Sugars and Organic Acids by HPLC

A Beckman Coulter HPLC apparatus connected to an IR (K-2301) and UV detector (K-2501) (Knauer, Berlin, Germany) was used to perform the chromatographic separation. An Aminex HPX-87H column (Bio-Rad, Hercules, CA, USA) was used to analyze the 20 μL samples collected during the GIT process before and after each phase (oral, gastric, in-testinal, and dialysis) after they had been filtered through a 0.45 μm cellulose acetate membrane. The column was operated at 40 °C with 5 mM H_2_SO_4_ as the mobile phase at a constant flow of 0.6 mL min^−1^ for 30 min. Clarity software, version 5.0.5.98 (DataApex, Prague, Czech Republic) was utilized for the data collection and processing. Calibration curves constructed for standards of fructose, glucose, and sucrose sugars, as well as citric, acetic, and succinic acids, were employed to quantify the individual sugars and organic acids, respectively, which were detected using an infrared and ultraviolet detector. All determinations were made in triplicate and results were expressed as mg of phenolic compounds per g DM.

### 2.8. Statistical Analysis

IBM^®^ SPSS^®^ Statistics software, Version 25 (IBM Corporation, New York, NY, USA) was used to perform the statistical analysis. The three biological experiments’ mean ± standard deviation was used to report the data. Using a one-way analysis of variance (ANOVA) and Tukey’s post hoc test for pairwise multiple comparison, differences across the various stages of GIT digestion were examined. At a threshold of *p* < 0.05, significant differences were considered. Additionally, Pearson’s correlation coefficient was calculated for the purpose of conducting the correlation analysis.

## 3. Results and Discussion

### 3.1. Effect of Simulated In Vitro Gastrointestinal Digestion on Bioactive Compounds

#### 3.1.1. Phenolic Compounds

**Figure 1:**
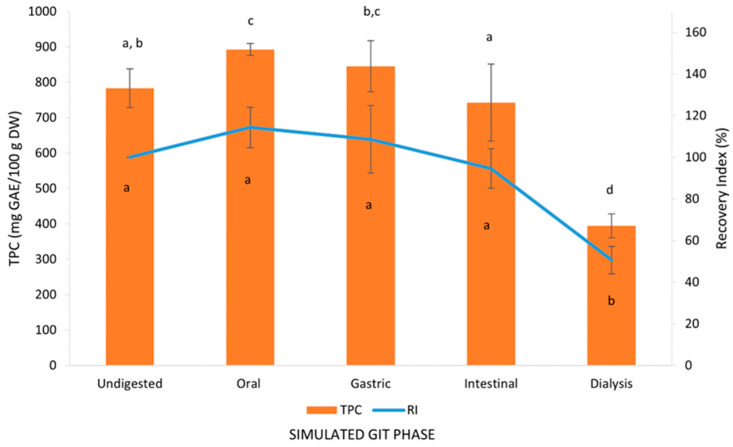
Phenolic compound recovery index (RI%) and total phenol content (TPC) during each stage of the *in vitro* gastrointestinal tract (GIT) simulation. The means of three separate calculations ± standard deviation are the results. Significant variations between GIT stages are indicated by values with different letters for each parameter (TPC or RI%).

It has been reported that phenolic compounds can be released along the GIT until they reach the intestine, where those that pass through the gut barrier can become available to exert their beneficial effects on health [41]. In our work (Figure 1), the level of TPC in the undigested sample (783.47 mg GAE/100 g DM) was shown to increase in the oral phase (892.41 mg GAE/100 g DM). However, no significant differences were observed in the gastric (845.21 mg GAE/100 g DM) and intestinal phases (742.94 mg GAE/100 g DM). In the last phase, TPC drastically decreased, with a concentration in the colon-available fraction of 394.64 mg GAE/100 g DM (*p* < 0.05). The percentage of polyphenols recovered in the oral, stomach, and intestinal phases were similar (114.36, 108.61 and 94.60%, respectively); however, in addition to TPC, the percentage of phenolic compounds in the colon-available fraction, i.e., after the absorption phase, was also significantly lower (50.61%). The increase in the amount of phenolic compounds recovered in the gastric phase, in contrast with the decrease in the intestinal phase (TPC RI%), has been previously described by other authors in several food matrices such as seed, stem and pomace of grape [42] and persimmon flours [43]. The enhanced recovery of phenolic compounds shown in the gastric phase could be due to the acid pH in the gastric phase of digestion, which promotes the release of bioactive compounds that are bound to different nutrients in food, such as fiber, proteins or carbohydrates, while the reduction after intestinal digestion was probably influenced by different factors, including, as follows: (i) the mild alkaline pH conditions present in the small intestine to which these compounds are very sensitive, leading to the degradation or transformation of dietary polyphenols; (ii) interactions with other components in the diet, such as proteins, carbohydrates or minerals, which impair the availability of phenolic compounds for absorption; (iii) chemical reactions resulting in the formation of other phenolic by-products; and (iv) variations in the molecular structure due to the action of enzymes, which may cause a reduction in their solubility [44].

Bioaccessibility, in the context of this study, is the amount of polyphenols ingested via food, released and solubilized, that are available for absorption in the gut after digestion [45]. The BI, according to Equation (1), was 45.65%, which means that nearly half of the digested sample in the small intestine can end up in the bloodstream, exerting its bioactive beneficial effect on the organism. In this respect, during GIT digestion, phenolic compounds in OLE could have undergone different changes that could have impaired their absorption, such as modifications of their chemical structure, increases or decreases in solubility, and interactions with other compounds [36]. Likewise, the compounds that remain in the fraction that are available for the colon can be metabolized by colonic bacteria, transforming dietary polyphenols into simple phenolic compounds, which can give rise to more biologically active metabolites [46]. The BI of TPC obtained in our work is in line with those previously reported by other authors on olive pomace (51.39%), persimmon flour (51.50%), and melon peel flour (67.51%) [18,41,43].

Regarding the phenolic profile, sixteen individual phenolic compounds were identified, thirteen of which were quantified before and after GIT digestion (Table 1). In relation to the digestion effect, it was observed that the different phases affected the stability and release of phenolic compounds from the food matrix. In general, there was a similar trend for most compounds, showing an increase in the oral and/or gastric phase and a decrease in the intestinal phase. These results suggest that gastric digestion enhances the release of phenolic compounds, probably due to enzymatic activity and/or the acidic environment that could facilitate the breaking of bonds within the dietary components of OL (proteins and fiber). Similarly, it was observed that intestinal digestion causes a decrease in phenolic acids, which could be explained by the instability of these compounds in alkaline conditions, by the formation of complexes between these compounds and others in the diet (metal ions, proteins and/or fiber), and/or by the interaction with bile salts [43].

As can be seen in Table 1, oleuropein, luteolin-7-O-glycoside and protocatechuic acid were the most abundant compounds in the undigested sample (181.07, 30.86 and 29.93 mg/100 g DM, respectively), while only hydroxytyrosol showed a significant increase in the intestinal phase and the highest percentage of recovery in this step (260.42%) (*p* < 0.05). Rocchetti et al. [47] reported similar changes during the *in vitro* gastrointestinal digestion of “Picual” extra virgin olive oil, where a reduction in oleuropein in the gastric and intestinal phase was observed, while hydroxytyrosol showed the opposite trend. The behavior of these two compounds could be due to the fact that oleuropein is hydrolyzed by enzymatic action mainly in the intestinal phase, yielding hydroxytyrosol, a degradation product of oleuropein [48].

It should be noted that in all digestion steps, TPC values (determined by the Folin–Ciocalteu method) were higher than the sum of phenolic compounds quantified by HPLC (Table 1). These results may be due to the following factors: (i) an overestimation of the TPC due to the low specificity of the Folin–Ciocalteu reagent; (ii) the non-quantitation of identified phenolic compounds by HPLC; and (iii) the non-identification of phenolic compounds [49]. Nevertheless, a high correlation was shown between TPC and the sum of individual phenolic compounds throughout the *in vitro* gastrointestinal digestion (*r* = 0.902), which indicates that, despite the remarkable differences observed between them, the behavior of these parameters followed a similar trend (Table S1).

In relation to the recovery of individual phenolic compounds, it can be observed in Table 2 that the RI increased in the oral and/or gastric phase, with the exception of gallic acid and oleuropein, which decreased slightly. However, in contrast, the RI of hydroxytyrosol (260.42%), 4-hydroxybenzoic (164.79%) and ferulic acid (145.51%) increased in the intestinal phase, representing some of the most accessible compounds that can be absorbed into the bloodstream (78.24, 97.86, and 97.13%, respectively). In this regard, nine out of the thirteen phenols presented a significantly high BI (between 78 and 98%). The present findings suggest that most OL phenols decreased throughout the digestion process while maintaining a relatively stable bioaccessibility. In agreement with this pattern, Ribeiro et al. [18] obtained similar recovery results in a study on olive pomace pulp-enriched powder, with a BI of tyrosol and caffeic acid of 63.06 and 87.68%, respectively. However, in contrast to our work, the authors found that hydroxytyrosol showed a greater recovery in the gastric and intestinal step, and a BI of 45.80%.

These highly bioaccessible phenolic compounds could be absorbed from the gut into the bloodstream to exert their effects on specific tissues or organs. Among the different compounds, tyrosol, hydroxytyrosol and oleuropein stand out for their association with the prevention of cardiovascular disease due to their ability to prevent the oxidation of low-density lipoproteins. Additionally, tyrosol has been associated with neuroprotective and anti-osteoporosis effects. Hydroxytyrosol has been associated with anti-tumor effects and protection against atherosclerosis and diabetic neuropathy. Oleuropein is known to contribute to the prevention of obesity problems by improving lipid metabolism and was shown to have a protective effect on enzymes and cell death in hypertensive patients with cancer [50,51].

It is also worth noting the influence of the gut microbiota on the bioavailability of phenolic compounds in the colon through biotransformation reactions. These compounds, by acting as a substrate for the microbiota, could promote the growth and proliferation of certain beneficial bacteria [51], as well as lead to more biologically active metabolites by their transformation into simple phenols [52]. In this study, it was observed that the main compounds available in the colon were hydroxytyrosol (56.94%), luteolin-6-glucoside (58.23%) and apigenin-7-glucoside (48.73%).

#### 3.1.2. Soluble Sugars and Organic Acids

The main soluble sugars present in the OL samples (Table 1) were fructose, glucose, and sucrose, showing values of 6.40, 4.38 and 2.49 mg/g DM, respectively, in the undigested samples. All three showed the same behavior, with an initial increase in the oral and gastric phases, although this was significant only for sucrose (*p* < 0.05), and a significant decrease in the three sugars in the intestinal phase, with respect to the gastric phase (*p* < 0.05). While fructose and glucose were not detected in the colon-available fraction, sucrose was found in a significantly low amount (*p* < 0.05), with an RI of 34.40%.

Similarly, regarding the percentage of soluble sugar recovered (Table 2), fructose and glucose exhibited a similar RI in the oral and gastric digestion phases (*p* > 0.05), while sucrose were significant higher in the gastric phase (*p* < 0.05). However, in the intestinal phase, a decrease was observed (*p* < 0.05), with sucrose presenting the highest value (163.70%), followed by fructose (77.65%) and glucose (66.09%). Despite the decrease in RI observed in the last step of digestion, the bioaccessibility of fructose, glucose and sucrose was high, with values of 97.48, 96.05 and 78.94%, respectively. These enhanced RI values might reflect the release of the compounds from the OL through the action of pH and digestive enzymes, which may result in the isomerization of sugars, such as glucose to fructose, explaining the higher RI of the latter [18]. In this regard, fructose has many advantages over glucose, including a low glycemic index, representing an interesting choice for diabetics. Fructose has been associated with improved performance during exercise when combined with glucose [41]. However, the limited impact of fructose on satiety may be a drawback in foods containing this sugar [53].

Glucose, fructose, mannitol, sucrose, galactose, and inositol are the main soluble carbohydrates found in olive leaf; the relative proportions differ according to the season. In this respect, a rise in glucose and a fall in sucrose has been reported in spring time [27,54], a fact that was observed in the OL samples used in this work. Likewise, the principal soluble sugar detected in olive pulp was shown to be glucose, followed by fructose and mannitol, while sucrose was present in very low concentrations [55]. Supporting this, other authors have identified glucose and fructose in liquid-enriched and pulp-enriched powder from olive pomace [18,39]. However, the recovery of both sugars in the intestinal digestion step was different in relation to our results; that is, the recovery was higher in pulp-enriched powder and lower in liquid-enriched powder than that in our work because the bioaccessibility of both sugars in the olive pomace fractions was considerably lower than that in our study.

Succinic acid, citric acid and acetic acid were the principal organic acids detected in OL (Table 1). In contrast to soluble sugars, organic acids were not significantly affected through the GIT; the exception was citric acid, the concentrations of which decreased in the intestinal phase (*p* < 0.05). Furthermore, none of the three acids were detected in the colon-available fraction. The recovery of succinic and acetic acid was highly stable throughout the different phases of GIT digestion (*p* > 0.05), whereas the RI of citric acid was halved in the intestinal step (50.03%). However, the BI was close to 100% for the three acids.

According to several authors, olives have a common organic acid profile [56,57]; the most relevant are malic, citric, succinic and oxalic acids. Among these, citric and succinic acids were the most abundant in OL in this work. Citric acid plays a crucial function in energy metabolism and macromolecule biosynthesis in the mitochondrial matrix, whereas succinic acid has been shown to be effective in reducing metabolic disorders associated with obesity. Similarly, succinate, found in living organisms in its succinate anion form, is considered a primary cross-feeding metabolite of the gut microbiota, as it is produced by primary fermenters and then consumed by secondary fermenters [41]. In addition to these acids, which are usually found in olives, others studies have described the presence of acetic acid in olive leaf [20,58].

It can be concluded that the high bioaccessibility of soluble OL sugars and organic acids at different stages of GIT indicates their potential as a functional ingredient rich in health-promoting compounds.

### 3.2. Effect of In Vitro Gastrointestinal Digestion on the Antioxidant Activity of OL

In this work, two methodologies based on different chemical mechanisms were used to assess the *in vitro* antioxidant activity of OL during GIT: electron transfer (ABTS) and hydrogen atom transfer (ORAC).

The antioxidant bioactivity of polyphenols has been widely related to their presence in plant matrices. Indeed, the correlation coefficients (*r*) between TPC and the antioxidant activity were 0.702 (with the ABTS assay) and 0.787 (with the ORAC assay). These values suggest that these compounds contributed greatly to the antioxidant activity.

**Figure 2:**
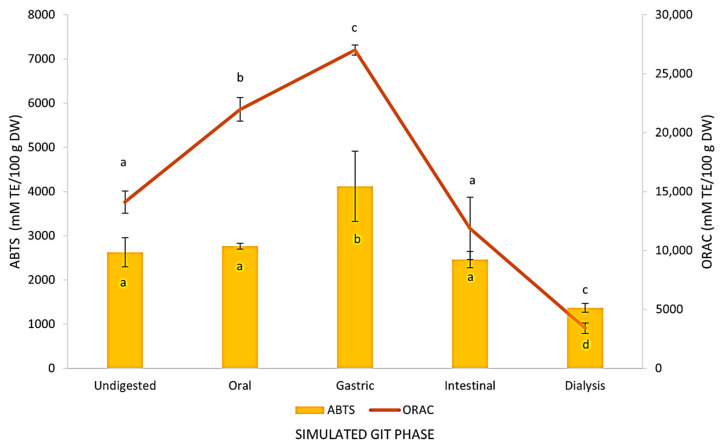
ABTS and ORAC techniques were used to evaluate antioxidant activity at each stage of the *in vitro* gastrointestinal tract (GIT) simulation. The means of three separate calculations ± standard deviation are the results. Significant variations between GIT phases are indicated by values with different letters within each method.

According to our results, the antioxidant activity of OL was affected by the simulated GIT in varying degrees, depending also on the assay performed, i.e., ABTS or ORAC. As can be seen in Figure 2, the ABTS values were not significantly different throughout the different phases of GIT, except for the gastric phase, where a significant increase was observed (4119.23 mM TE/100 g DM), and the dialysis phase, where a significant decrease was observed (1368.80 mM TE/100 g DM). However, ORAC values significantly increased until the gastric step (27009.97 mM/100 g DM) and subsequently decreased until the final absorption step (3407.96 mM TE/100 g DM). The lower values of the ABTS assay with respect to the ORAC assay, as shown in Figure 2, are probably due to the higher molecular weight of the ABTS radical than the ORAC molecule, which may reduce the reaction rate of the former, thereby making the measurement of antioxidant capacity using the ORAC method more accurate [40]. Despite the observed differences, a significant correlation between the ABTS and ORAC assays in OL was found (*r *= 0.862) (Table S1).

The RI of the antioxidant activity was also calculated for both methods (Table 2), showing the same trend, i.e., the highest RI was observed in the gastric phase (157.36 and 192.21% for ABTS and ORAC, respectively), a slight decrease was seen in the intestinal phase (94.88 and 85.22%, respectively), and a more pronounced fall was observed in the last phase of dialysis (52.71 and 24.11%, respectively). However, in terms of BI, the ORAC assay presented a significant higher value (70.03%) than the ABTS (44.34%).

As mentioned above, the higher antioxidant activity in the gastric phase can be explained by a higher release of phenolic compounds due to the action of acid pH and enzymatic activity, while the lower antioxidant activity in the intestinal phase may be due to the degradation or transformation of dietary polyphenols into other compounds in the small intestine [39].

Similar antioxidant values have been previously reported in the literature; for instance, in studies on *Q. ilex* leaf (ABTS 472.97 and ORAC 610.46 mM TE/g DW) and pomegranate peel flour (ABTS 66.12 mg TE/g and ORAC 183.22 ug TE/g), both matrices were shown to have the highest values in the gastric phase [36,44]. Likewise, a reduction in antioxidant activity in the intestinal phase was observed in studies on rosemary extract (9.60%) and crisphead lettuce (35.80%) when evaluated by the ORAC and ABTS methods, respectively [52,59].

## 4. Conclusions

In this research, the recovery and bioaccessibility of bioactive compounds from ground olive leaf (OL) in the gastrointestinal tract (GIT) were studied. The results showed that, in general, *in vitro* digestion had a significant effect on the stability of phenolic compounds and antioxidant activity of OL, especially in the last phases of the GIT, i.e., the intestinal and absorption phases. However, TPC (45.65%), ABTS (44.34%) and ORAC (70.03%) showed high bioaccessibility; therefore, these compounds have high potential of rendering health-related benefits.

Despite these promising findings, this study has certain limitations. First, the *in vitro* digestion model, while widely used, does not fully replicate the complexity of human digestion. Furthermore, the study focused on bioaccessibility but did not assess actual bioavailability, which requires further *in vivo* studies. Additionally, the potential impact of the food matrix in novel food formulations on bioaccessibility was not explored. Future research should explore *in vivo* models to validate these findings and evaluate the potential applications of ground olive leaves in functional food formulations.

Overall, our results suggest that olive leaf constitutes a good source of phenolic compounds and other sources of carbon (such as sugars or acids) with health-exerting antioxidant effects, that, together with the fiber present in OL, could be applied in the development of functional foods or ingredients using the circular economy approach.

## Figures and Tables

**Table 1:** Concentration of bioactive compounds identified and quantified by HPLC in ground olive leaf before (undigested samples) and after *in vitro* gastrointestinal digestion (oral, gastric, intestinal and dialysis).

Bioactive Compounds	Gastrointestinal Phase				
	Undigested	Oral	Gastric	Intestinal	Dialysis
Phenolic compounds					
Gallic acid	17.94 ± 1.05 ^a^	16.46 ±1.03 ^a^	16.64 ± 0.77 ^a^	6.46 ± 0.03 ^b^	ND
Protocatechuic acid	29.93 ± 1.17 ^a^	37.51 ± 0.50 ^b^	19.24 ± 1.02 ^c^	8.96 ± 0.10 ^d^	ND
Hydroxytyrosol	3.01 ± 0.84 ^a^	9.13 ± 1.41 ^c^	4.61 ± 1.41 ^a,b^	7.61 ± 0.74 ^b,c^	1.68 ± 0.06 ^a^
4-hydroxybenzoic	7.45 ± 0.69 ^a^	14.13 ±0.22 ^b^	14.76 ± 0.67 ^b^	12.36 ± 2.73 ^a,b^	ND
Tyrosol	19.32 ± 4.32 ^a^	39.93 ± 1.64 ^b^	42.97 ± 5.95 ^b^	9.10 ± 0.53 ^a,c^	0.55 ± 0.03 ^c^
Vanillic acid	NQ	NQ	NQ	NQ	ND
Caffeic acid	3.41 ± 0.72 ^a,b^	5.84 ± 1.79 ^b^	5.34 ± 0.08 ^b^	1.31 ± 0.26 ^a^	ND
Vanillin	4.62 ± 1.31 ^a,b^	10.64 ± 0.39 ^a,b^	14.67 ± 6.09 ^a^	4.05 ± 0.08 ^a,b^	UQ
Feluric acid	8.56 ± 1.64 ^a^	15.62 ± 3.63 ^a,b^	17.82 ± 0.52 ^b^	12.24 ± 0.40 ^a,b^	ND
Isoferulic acid	NQ	NQ	NQ	ND	ND
Myricetin	NQ	NQ	NQ	NQ	NQ
Luteolin-6-Glycoside	11.69 ± 0.74 ^a,b^	19.11 ± 6.25 ^a^	12.68 ± 0.66 ^a,b^	11.74 ± 0.68 ^a,b^	6.78 ± 0.01 ^b^
Luteolin-7-O-Glycoside	30.86 ± 4.48 ^a^	34.01 ± 5.04 ^a^	27.15 ± 9.64 ^a^	12.42 ± 1.24 ^b^	11.08 ± 0.42 ^b^
Apigenin-7-Glycoside	2.90 ± 0.54 ^a^	2.90 ± 0.54 ^a^	2.47 ± 0.52 ^a^	1.85 ± 0.48 ^a^	1.38 ± 0.19 ^a^
Apigenin	3.09 ± 0.24 ^a^	3.82 ± 1.02 ^a^	3.01 ± 0.13 ^a^	3.08 ± 0.03 ^a^	0.64 ± 0.05 ^b^
Oleuropein	181.07 ± 5.44 ^a^	162.25 ± 5.89 ^a^	82.07 ± 18.71 ^b^	54.79 ± 8.74 ^b^	1.86 ± 0.20 ^c^
Total by HPLC	323.86 ± 23.20 ^a^	371.37 ± 29.40 ^a^	263.42 ± 46.16 ^a,b^	144.63 ± 15.22 ^b,c^	24.96 ± 1.78 ^c^
TPC by Folin–Ciocalteu	783.47 ± 54.81 ^a,b^	892.41 ± 17.02 ^c^	845.21 ± 72.22 ^b,c^	742.94 ± 108.89 ^a^	394.64 ± 33.98 ^d^
Soluble sugars					
Fructose	6.40 ± 0.65 ^a,b^	7.57 ± 0.98 ^a^	8.36 ± 0.89 ^a^	5.32 ± 0.97 ^b^	ND
Glucose	4.38 ± 0.49 ^a^	4.55 ± 0.14 ^a^	4.55 ± 0.55 ^a^	3.12 ± 0.53 ^b^	ND
Sucrose	2.49 ± 0.38 ^a^	4.59 ± 0.28 ^b^	7.83 ± 0.42 ^c^	4.07 ± 0.24 ^b^	0.86 ± 0.08 ^d^
Organic acids					
Citric acid	17.55 ± 1.05 ^a^	18.60 ± 0.92 ^a^	17.62 ± 1.08 ^a^	9.07 ± 2.17 ^b^	ND
Succinic acid	21.47 ± 4.16 ^a^	21.79 ± 5.06 ^a^	16.94 ± 3.51 ^a^	15.92 ± 5.81 ^a^	ND
Acetic acid	5.58 ± 0.51 ^a^	6.13 ± 1.56 ^a^	6.39 ± 0.71 ^a^	5.46 ± 0.39 ^a^	ND

Values are expressed as mean of three determinations ± standard deviation. Concentrations that differ significantly (*p <* 0.05) are indicated by values in the same row with different superscript characters. NQ: not measurable. ND: not found. UQ: below the limit of quantification. Units: soluble sugars: mg/g DM; organic acids: mg/g DM; phenolic compounds: mg/100 g DM; TPC: mg GAE/100 g DM.

**Table 2:** Recovery index (RI%) and bioaccessibility index (BI%) of bioactive compounds throughout *in vitro* gastrointestinal digestion.

Bioactive Compounds	Recovery Index (%)				Bioaccessibility Index (BI%)
	Oral	Gastric	Intestinal	Dialysis	
Phenolic compounds					
Gallic acid	91.98 ± 11.34 ^a^	93.05 ± 9.83 ^a^	35.98 ± 1.73 ^b^	UQ	96.12 ± 1.14 ^a,b^
Protocatechuic acid	125.51 ± 6.69 ^a^	64.25 ± 0.96 ^b^	29.91 ± 1.44 ^c^	UQ	97.20 ± 0.81 ^a,b^
Hydroxytyrosol	303.33 ± 47.14 ^a^	168.34 ± 25.93 ^a,b^	260.42 ± 50.09 ^a^	56.94 ± 13.75 ^b^	78.24 ± 1.09 ^d,e^
4-hydroxybenzoic	190.51 ± 13.43 ^a^	199.08 ± 25.55 ^a^	164.79 ± 22.94 ^a^	UQ	97.86 ± 1.05 ^a^
Tyrosol	210.76 ± 38.58 ^a^	224.38 ± 19.32 ^a^	47.89 ± 7.75 ^b^	2.87 ± 0.28 ^b^	93.97 ± 0.40 ^a,b,c^
Caffeic acid	173.17 ± 12.56 ^a^	164.38 ± 40.28 ^a^	38.87 ± 0.58 ^b^	UQ	88.79 ± 3.01 ^a,b,c,d^
Vanillin	231.52 ± 7.69 ^a,b^	319.57 ± 132.20 ^a^	91.33 ± 23.73 ^a,b^	UQ	87.65 ± 0.22 ^a,b,c,d^
Feluric acid	182.33 ± 7.50 ^a^	212.06 ± 34.55 ^a^	145.51 ± 23.54 ^a^	UQ	97.13 ± 0.66 ^a,b^
Luteolin-6-Glycoside	165.16 ± 33.17 ^a^	108.48 ± 12.00 ^a^	100.45 ± 0.63 ^a^	58.23 ± 3.52 ^a^	42.04 ± 3.14 ^f^
Luteolin-7-O-Glycoside	111.37 ± 16.08 ^a^	89.12 ± 15.39 ^a,b^	40.79 ± 8.18 ^b,c^	36.28 ± 6.39 ^c^	10.28 ± 2.23 ^g^
Apigenin-7-Glycoside	93.10 ± 9.75 ^a^	84.483 ± 17.068 ^a^	63.79 ± 17.07 ^a^	48.73 ± 4.63 ^a^	0.91 ± 0.012 ^g^
Apigenin	121.63 ± 20.84 ^a^	97.39 ± 13.45 ^a^	100.42 ± 9.16 ^a^	21.16 ± 4.21 ^b^	79.03 ± 2.28 ^c,d,e^
Oleuropein	89.61 ± 0.55 ^a^	45.18 ± 8.99 ^b^	30.21 ± 3.93 ^b^	1.02 ± 0.15 ^c^	96.55 ± 0.94 ^a,b^
Total by HPLC	114.61 ± 1.23 ^a^	80.73 ± 11.98 ^b^	44.55 ± 2.13 ^c^	7.71 ± 0.00 ^d^	82.68 ± 0.84 ^b,c,d,e^
TPC by Folin–Ciocalteu	114.36 ± 9.72 ^a^	108.61 ± 16.15 ^a^	94.60 ± 9.52 ^a^	50.61 ± 6.60 ^b^	45.65 ± 13.03 ^f^
Soluble sugars					
Fructose	118.20 ± 8.48 ^a^	130.88 ± 11.86 ^a^	77.65 ± 11.60 ^b^	UQ	97.48 ± 0.66 ^a,b^
Glucose	104.79 ± 9.91 ^a^	104.55 ± 13.40 ^a^	66.09 ± 7.51 ^b^	UQ	96.05 ± 0.95 ^a,b^
Sucrose	184.61 ± 11.15 ^a^	314.62 ± 16.89 ^b^	163.70 ± 9.73 ^a^	34.40 ± 3.09 ^c^	78.94 ± 2.18 ^c,d,e^
Organic acids					
Citric acid	106.40 ± 11.11 ^a^	100.45 ± 5.66 ^a^	50.03 ± 9.55 ^b^	UQ	98.63 ± 0.58 ^a^
Succinic acid	101.02 ± 4.71 ^a^	70.28 ± 15.47 ^a^	83.76 ± 21.68 ^a^	UQ	99.05 ± 0.28 ^a^
Acetic acid	108.73 ± 19.11 ^a^	114.77 ± 10.92 ^a^	98.23 ± 6.73 ^a^	UQ	97.48 ± 0.27 ^a,b^
Antioxidant activity					
ABTS	106.21 ± 12.08 ^a^	157.36 ± 29.73 ^b^	94.88 ± 16.37 ^ac^	52.71 ± 8.33 ^c^	44.34 ± 1.38 ^f^
ORAC	155.95 ± 3.99 ^a^	192.21 ± 15.39 ^a^	85.22 ± 25.12 ^b^	24.11 ± 1.61 ^c^	70.03 ± 8.71 ^e^

Values are expressed as mean of three determinations ± standard deviation. For RI (%), concentrations that are significantly different (*p <* 0.05) are indicated by numbers in the same row with different superscript letters. Significant differences (*p <* 0.05) across bioactive molecules are shown by distinct superscript letters for BI (%) throughout the column. UQ: below the limit of quantification.

## Data Availability

The original contributions presented in the study are included in the article/Supplementary Material, further inquiries can be directed to the corresponding author.

## Supplementary Materials

The following supporting information can be downloaded at: https://www.mdpi.com/article/10.3390/foods14040563/s1, Table S1: Pearson´s correlation coefficient between TPC and the sum of individual phenolic compounds and, ABTS and ORAC assays.
