# Supplementary material for: Bioactive Potential of Olive Leaf By-Product Throughout In Vitro Gastrointestinal Digestion
Source: Foods. 2025 Feb 8;14(4):563. doi: 10.3390/foods14040563 (PMC11853783; doi:10.3390/foods14040563)
Supplement: Supplementary file 1 [file foods-14-00563-s001.zip › foods-3443304-supplementary.pdf]

Table S1: Pearson's correlation coefficient between TPC and the sum of individual phenolic compounds and, ABTS and ORAC assays.

	**ABTS**	**ORAC**	**TPC**	**Sum of individual phenolic compounds**
**ABTS**	1	0.86	-	-
**ORAC**	0.86	1	-	-
**TPC**	-	-	1	0.9
**Sum of individual phenolic compounds**	-	-	0.9	1
